# Is the clinical pattern of pediatric celiac disease changing? A thirty-years real-life experience of an Italian center

**DOI:** 10.1186/s13052-021-01183-5

**Published:** 2021-12-14

**Authors:** Melissa Pedretti, Francesca Sbravati, Davide Allegri, Flavio Labriola, Virginia Lombardo, Enzo Spisni, Chiara Zarbo, Patrizia Alvisi

**Affiliations:** 1 Specialty School of Paediatrics - Alma Mater Studiorum, University of Bologna, Bologna, Italy; 2 Pediatric Gastroenterology Unit, Maggiore Hospital, Pediatric Department, Bologna, Italy; 3 Department of Clinical Governance and Quality, Bologna Local Healthcare Authority, Bologna, Italy; 4 Department of Biological, Geological and Environmental Sciences, University of Bologna, Bologna, Italy

**Keywords:** Pediatric celiac disease, Clinical pattern, Age at diagnosis, Clinical trend over time

## Abstract

**Objectives:**

Clinical presentation of pediatric celiac disease (CD) is heterogeneous and ever-evolving. Our aim is to highlight its changes throughout the years.

**Methods:**

Data about clinical presentation of CD in children diagnosed between 1990 and 2020 at the CD Center of Maggiore Hospital, Bologna, were collected. Patients were stratified into groups based on the date [P1 (1990–2011), P2 (2012–2020)] and age [G1 (< 2 years), G2 (2–5), G3 (6–11), G4 (12–18)] at diagnosis, then investigated by comparing CD clinical presentation in different periods and ages.

**Results:**

1081 children were selected. Mean age at diagnosis increases from 5.9 to 6.6 years from P1 to P2. Gastrointestinal Symptoms (GIs) are predominant, with a decline of diarrhea (47%VS30%) and an increase of constipation (4%VS19%) (*p* < 0.001). Among Extraintestinal symptoms (EIs) a decrease of anemia (76%VS43%, *p* = 0,001) is observed. Failure to Thrive (FTT) is stable throughout the years (*p* = 0.03), while screenings show a trend of increment (19%VS23%). GIs’ frequency decline from G1 to G4 (*p* = 0,001), with reduction of diarrhea (*p* < 0.001), and rise of recurrent abdominal pain (*p* = 0,02). EIs are more frequent at older ages, FTT in younger patients.

**Conclusions:**

Changes in clinical presentation of CD have occurred in the last 30 years. We observe a reduction of severe and classic gastroenterologic symptoms and a rise of atypical ones, together with a growth of serological screenings and higher age at diagnosis. Awareness about CD clinical trends is crucial for a proper approach and early diagnosis.

## Introduction

Celiac disease (CD) is an immune-mediated gluten-dependent enteropathy in genetically predisposed individuals, with an estimated prevalence of 1% [1, 2]. The only effective treatment is lifelong strict Gluten-Free-Diet (GFD) [1]. However, this condition is often under-diagnosed because of heterogeneity of signs and symptoms at presentation, or because it is completely silent [3]. Serum-prevalence data suggest that for each clinically diagnosed patient, an average of 5–10 seropositive individuals remains undiagnosed, usually due to atypical symptoms, minimal or even absent [3]. The understanding of epidemiological trends of CD in the early stages of life can guide the optimal approach to the rising burden of such an heterogeneous and widespread condition and its early diagnosis.

The pediatric clinical presentation of CD is extremely variable, characterized by intestinal and/or extraintestinal signs and symptoms variously combined. The most frequent gastrointestinal symptoms (GIs) include diarrhea, constipation, recurrent abdominal pain (RAP), bloating, and vomiting [4]. CD can also present with extra-intestinal symptoms (EIs) and signs hardly linkable to the disease, including anemia, delayed puberty, headache, recurrent dermatitis, dental enamel defects [5] and failure to thrive (FTT). Moreover, there are a number of asymptomatic patients with increased risk of CD due to first degree familiarity or presence of comorbidities [6], who are diagnosed through screening programs. The clinical presentation also changes according to age [4].

The aim of this study is to analyze how CD clinical presentation in children has changed in an Italian Pediatric referral Centre in the last 30 years, according to age and period at diagnosis, before or after 2012 guidelines for pediatric CD diagnosis [6].

## Methods

We conducted an observational retrospective study in a population of children (age ⋜18 years old) diagnosed with CD from January 1990 to March 2020 at the Pediatric Gastroenterology referral Centre for Celiac Disease of Maggiore Hospital in Bologna. Personal and clinical data were extracted from clinical reports of each patient and collected: sex, age at diagnosis, clinical presentation, familiar history of CD, comorbidities, and patients identified by routinary screening.

All enrolled patients were diagnosed according to criteria from effective European CD guidelines at the time of medical examination. Up to 2011 small-bowel mucosal biopsies during esophagogastroduodenoscopy (EGDS) were required to confirm CD diagnosis [4]. According to 2012 guidelines from the European Society for Pediatric Gastroenterology, Hepatology and Nutrition (ESPGHAN), the histological assessment may be omitted in symptomatic patients who have IgA anti-transglutaminase antibodies (TGA-IgA) ⋝10 times above the upper limit of normal (ULN), verified by endomysial antibodies (EMA-IgA) positivity and HLA-DQ2 and/or HLA-DQ8 heterodimer [6]. Furthermore, the most recent update of the 2020 ESPGHAN guidelines provides the possibility of diagnosing CD in the absence of symptoms and predisposing HLA haplotype [1].

Our population was stratified into two groups based on the time at diagnosis: period 1 (P1) if patients were diagnosed between January 1990 and December 2011, period 2 (P2) between January 2012 and March 2020. These intervals were settled in order to have comparable groups and they consider the important changes in pediatric CD diagnosis after the 2012 ESPGHAN guidelines publication. Furthermore, we divided patients into 4 subgroups based on the age at diagnosis: group 1 (G1) age < 2 years old; group 2 (G2) age 2–5 years; group 3 (G3) age 6–11 years; group 4 (G4) age 12–18 years old.

Patients were investigated by comparing clinical presentation of CD at diagnosis. We identified four categories of symptoms: **GIs**, which include diarrhea, RAP, constipation, vomiting/gastroesophageal reflux (GER), stomatitis and bloating; **EIs**, including anemia, dental enamel defects, neurological symptoms (headache, fainting/syncope, epilepsy), skin/appendages involvement (rashes, nail and hair alterations*);* **FTT**, defined as a significant height or weight deceleration compared with the reference rate for age and sex or compared with expected height based on parental height; **screening** group (first-degree familiarity for CD, increased risk due to comorbidities such as autoimmune associated conditions, chromosomal abnormalities, or selective IgA deficiency, and patients with neither obvious symptoms nor risk factors). Each patient may have been assigned to more than one group.

The frequency of symptoms at presentation has been studied in relation either to the year of diagnosis and the age at the time of diagnosis. The study was approved by the local review board.

### Statistical analyses

Continuous variables were expressed as means and standard deviations (SD), while categorical variables were presented as frequencies and percentages. Comparisons of continuous variables between more than two groups were carried out using the Kruskal-Wallis tests while the association between categorical variables was tested by means of the Fisher exact test or χ2, as appropriate. Tests were 2-sided and a *P* < 0.05 was considered significant. Analyses were performed with STATA software 15.

## Results

### Population

We evaluated 1081 celiac children. 517 are included in P1 and 564 in P2. Concerning age groups, 13% (*n* = 141) of patients are diagnosed within the first 2 years of life, 40,7% (*n* = 440) from 2 to 5 years old, 37% (*n* = 401) from 6 to 11, and 9,2% (*n* = 99) over 12 years old. Overall, the mean age at diagnosis is 6,3 (SD ± 3,75) years old, median age is 5,6 [range 0,8–18] years old. Female/male ratio is 1,9:1, with 66%, (*n* = 718) of females. Overall, the most frequent clinical presentation is represented by GIs (44%), followed by FTT (24%) and EIs (15%). Finally, 412 (38%) children are detected by screening, of whom 226 (21%) patients with no risk factors, 115 (11%) with first-degree familiarity for CD, and 71 (6%) present associated conditions.

### Clinical trends in relation to periods

Symptoms and demographic characteristics at presentation according to the 2 considered periods are reported in detail in Table 1**.**

**TABLE 1:** Clinical presentation of celiac disease in relation to the two considered periods

	P1 (1990–2011) *n* = 517 n (%)	P2 (2012–2020) *n* = 564 n (%)	***p*** value	Total 1081 n (%)
**Symptoms**				
*GASTROINTESTINAL*	*182 (35%)*	*294(52%)*	*p < 0.001*	*476 (44%)*
Diarrhea	85 (47%)	88 (30%)	*p* < 0.001	173 (36%)
Recurrent abdominal pain	87 (48%)	150 (51%)	*p* = 0.49	237 (50%)
Constipation	8 (4%)	57 (19%)	*p* < 0.001	65 (14%)
Vomit / GER	23 (13%)	28 (10%)	*p* = 0.29	51 (11%)
Stomatitis / Aftosis	1 (1%)	10 (3%)	*P = 0.06*	11 (2%)
Bloating	2 (1%)	36 (12%)	*p* < 0.001	38 (8%)
*EXTRAINTESTINAL*	*72 (14%)*	*86(*15%)	*p = 0.54*	*158 (15%)*
Anemia	55 (76%)	37 (43%)	*p* < 0.001	92 (58%)
Neurologic symptoms	7 (10%)	21 (24%)	*p = 0.02*	28 (18%)
Skin	9 (13%)	25 (29%)	*p* = 0.01	34 (22%)
Dental Enamel Defects	1 (1%)	6 (7%)	*p* = 0.13	7 (4%)
*SCREENING*	*199(38%)*	*213(38%)*	*p = 0.81*	*412(38%)*
Absence of symptoms/risk factors	97 (49%)[19%]*	129 (60%)[23%]*	*p* = 0.09	226 (55%)[21%]*
1st degree familiarity	58 (29%)	57 (27%)	*p* = 0.61	115 (28%)
Autoimmune associated conditions	44 (22%)	27 (13%)	*p* = 0.01	71 (17%)
*FAILURE TO THRIVE*	*140(27%)*	*121 (21%)*	*P = 0.03*	*261 (24%)*
***Age Groups***				
G1 (< 2.0 years)	*94 (18%)*	*47 (8%)*	*p* < 0.001	*141 (13%)*
G2 (2.1–6.0 years)	*212 (41%)*	*228 (40%)*	*p = 0.85*	*440 (41%)*
G3 (6.1–12.0 years)	*163 (32%)*	*238 (42%)*	*p* < 0.001	*401 (37%)*
G4 (12.1–18 years)	*48 (9%)*	*51 (9%)*	*p = 0.89*	*99 (9%)*

[]* % on the total period’s sample

The mean age at diagnosis gradually increases from 5.9 years old (SD ± 3.82) in P1 to 6.6 (SD ± 3.65) in P2 (*p* < 0.05).

Over the 30 examined years we observe a significant decrease in children diagnosed < 2 years of age (18% in P1 vs 8% in P2, *p* < 0,001), and an increase in patients detected at 6–11 years old (32% in P1 vs 42% in P2, *p* < 0,001). Diagnosis at age 2–5 and 12–18 years old are statistically overlapping (*p* = 0,85 and *p* = 0,89, respectively) in the two periods. We found an higher prevalence among females.

**GIs** are predominant for frequency in P1 (35%) and P2 (52%). In detail, in both considered periods RAP is prevailing (48% in P1, 51% in P2), followed by diarrhea (47% in P1, 30% in P2), vomiting/GER (13% in P1, 10% in P2) and constipation (4% in P1, 19% in P2). Comparing the prevalence of each GIs in the 2 studied periods we find that diarrhea significantly decreases over the years (*p* < 0.001), while constipation and bloating increase (*p* < 0.001).

**EIs** are less frequent in both P1 (14%) and P2 (15%), without differences in the two periods (*p* = 0.54). Neurological associated symptoms are represented by headache in all patients of our sample. In P1 the most frequent EIs is anemia, which significantly decreases from P1 to P2 (76% vs 43%; *p* < 0.001), while neurological manifestations and skin involvement increase (*p* < 0,05).

Recurrence of **FTT** is stable throughout the years (27% in P1 vs 21% in P2, *p* = 0.03).

The overall percentage of patients identified by **screening** also remains stable over the 30 years. However, if we exclude from this group children who were screened because of a family history for CD (29% in P1 and 27% in P2) or comorbidities (22% in P1 and 13% in P2), we find that patients without any risk factor grow from 18,7% in P1 to 22,9% in P2, on the total sample. The difference doesn’t reach a statistical significance, but we observe a trend of increase in diagnosis from routinary screening programs.

### Clinical trends in relation to age at diagnosis

**Fig. 1:**
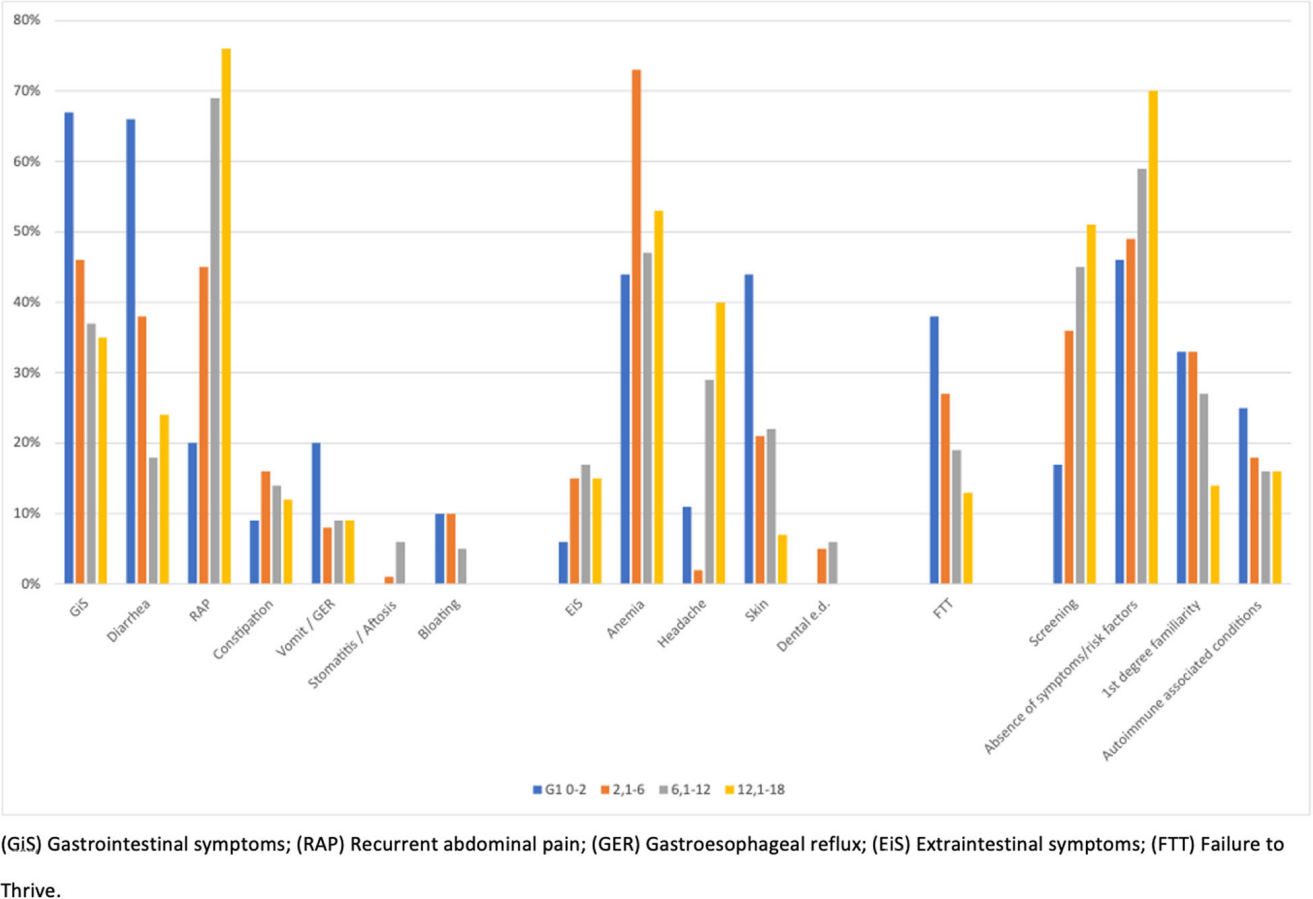
Symptoms related to age groups

Symptoms and demographic characteristics at presentation according to the 4 considered age groups are reported in Fig. 1.

**GIs’** frequency significantly decreases with the raise of age at diagnosis: G1 has the highest rate (67%), followed by G2 (46%), G3 (37%), and G4 (33%). In detail, there is a gradual decrease of diarrhea from G1 to G4 (66% vs 24%, *p* < 0.001), while RAP has an opposite trend (20% in G1 vs 75% in G4, *p* = 0,02). The frequency of constipation does not change in relation to the different age groups (*p* = 0.38). Finally, we observe a decrease in the incidence of vomiting (*p* < 0,001) and bloating (*p* = 0,01) from G1 to G4.

With regard to **EIs**, we observe a significantly lower frequency in G1 compared to other age groups (*p* = 0,02). Concerning anemia, G2 has the higher frequency among all age groups (*p* = 0,01). Neurological symptoms are lower in G2 than in all older age groups (*p* < 0.05).

The frequency of **FTT** gradually decreases with the raise of age at diagnosis (*p* < 0.05).

The frequency of **screenings** gradually increases from 17% in G1 to 51% in G4 (*p* < 0.05). With regard to patients with no obvious symptoms nor risk factors, their frequency is significantly higher in G4 (70%) than G1 (46%) and G2 (49%), (*p* < 0.05). On the contrary, in younger patients the frequency of CD diagnosis by family screening is higher than in the older age groups (G1 and G2 33% vs G4 14%; *p* < 0.05). There are no relevant differences between the age groups in relation to comorbidities.

## Discussion

Data presented in this research highlight significant changes in clinical presentation of pediatric CD in the last 30 years, in the wider population so far reported in the literature. Several smaller studies documented changes in the clinical pattern of pediatric CD [7–11] in the last decades. In particular, different authors have demonstrated a progressive increase of age at diagnosis throughout the years [7, 8, 11, 12]. We confirm a comparable trend, however, we report a lower mean age at diagnosis than both Northern and Southern Europe [8, 11, 13] and United States of America (USA) [7, 14]. In the last decade, the average age at diagnosis in our population is 6.6 years compared to over 9 years in other areas [8, 14]. These findings agree with previous studies conducted in the Mediterranean area, that found average ages of 5.5–6 years old [10, 12]. Different infant feeding practises could influence the age at diagnosis and clinical CD presentation. A possible explanation is that in Italy there is a higher intake of gluten in the first years of life. Indeed, according to the Food and Agricultural Organization (FAO), in Italy daily wheat supply quantity corresponds to 401.2 g/capita/day, whereas in Northern Europe and USA it is respectively 263.5 and 220.36 g/capita/day [15]. Moreover, in our geographical area gluten is introduced earlier in childrens’ diet. According to Lionetti E. et al. [16], a delayed gluten introduction, although it does not affect the onset of CD, is associated with a delayed onset of the disease. In second place, breastfeeding’s duration could influence the age of CD onset. In 2016 ESPGHAN stated that breastfeeding does not reduce the risk of CD in children [17], but several evidences speculate that prolonged breastfeeding delays the onset of symptoms [18, 19]. In Italy breastfed infants at 6 months of life are 38% while in Sweden and Norway they are respectively 61 and 71% [20]. The increased age at diagnosis of the last decade in our population is related to a significant decrease of patients < 6 years old, and a progressive rise in children with age 6–12 years. This may be explained by the expanding implementation of routinary screening programs in asymptomatic patients and the increasing awareness about atypical signs and symptoms of CD or Eis, mainly in older patients [21].

In our study GIs are the dominant symptoms that lead to CD diagnosis at all times (35–52%) increasing in our case series over the past 30 years, but fewer than in other countries [8], as reported in a Swedish study conducted between 1973 and 2013 (71–82%) [8].

GIs’ prevalence, mainly in younger children, may occur because they are the most striking symptoms and likely to be detected by parents and clinicians.

RAP remains the predominant GI symptom over the time and its frequency increases in older children, presumably because the pain has to be reported directly by the patient. In the European Union (EU) pediatric population, RAP has been recently identified as the most represented symptom in all Countries except for Italy [14], however, our results from a much larger sample, although monocentric, seem to align the Italian trend with the rest of EU countries. In accordance with our findings, a large British study of 13,971 children observe that RAP is reported in 11.8% of 6-years-old children [22], while it is less common at the age of 2 (3.8%) and 3 (6.9%). We should also notice that sometimes RAP is functional and does not relate to CD, as already suggested in the literature [23]. Unlike RAP, diarrhea and vomiting are declining throughout the ages in our sample, probably due to the improvement in diagnostic skills about these early, traditional and severe symptoms. On the other hand, we notice a rise of less common GIs like constipation and bloating. In accordance with these findings, in recent years constipation has been recognized as a symptom related to CD that needs to be investigated [24].

In the literature there are no univocal data about the course of EIs over time. In particular, a recent study [9] reports a steady trend of anemia, while other sources [8, 11] report a reduction in EIs over time. Overall, their prevalence is stable throughout the considered time in our population. Among EIs, anemia is prevailing in all periods but it’s frequency is declining in P2, probably due to a reduction of severe gastrointestinal forms of malabsorption in recent years. We may remember that even though anemia is commonly associated with severe mucosal atrophy, it can be the only sign of CD [25]. Furthermore, we observe an increase in neurological manifestations and skin/appendages involvement through the years. A better detection of non-classic presentations may be related to an improved primary-care pediatricians’ awareness, thanks to continuous educational programs. In Emilia Romagna, which has almost 18,000 celiac patients both adults and children [26] (4.459.000 inhabitants), a regular training is carried out by pediatric gastroenterologists to general pediatricians, in order to allow adequate prescriptions of blood test examinations for CD and an early referral to the specialist of suspected children. Moreover, since the only neurological manifestation in our sample is headache, we specify that in our hospital coexists a pediatric neuropsychiatry tertiary-level centre where many children are referred for this condition. EIs’ lower frequency in patients < 2 years old compared to other age groups, suggests a later onset of these symptoms. Anemia is more frequent at 2–6 years of age, and this may be a consequence of a delay in detecting severe forms of malabsorption in which anemia is the only sign.

We found no significant differences about FTT recurrence among the examined periods, while both in Northern Europe and USA a few studies report decreasing trends in the last years [7, 8, 11].

We observe a tendency of increase in patients identified from routinary screening programs in the last decade. This can be explained by the growing diffusion of screening practices for patients with a first-degree relative affected by CD, or even asymptomatic children without any risk factor. However, in our series, the patients identified through screening programs are predominantly older than 2, as blood tests are rarely performed before this age in the absence of symptoms.

There are some weaknesses in this research. It is a retrospective and monocentric study, even if our Centre is a referral one and our cohort is the largest among the published literature on the issue. Therefore, despite these limitations, we believe that our results are a helpful contribution to the understanding of current CD clinical presentation and give fundamental support to the existing literature.

In conclusion, we can assess that significant changes in clinical presentation of pediatric CD have occurred in the last 30 years. The average age at diagnosis has increased throughout the decades and it confirms to be lower compared to other Countries; this may be related to specific Italian environmental factors such as diet and breastfeeding practises. GIs represent the main symptoms at onset, mostly in younger patients, but we recently recorded a progressive reduction of severe and classic signs and symptoms and a rise of atypical clinical patterns. Furthermore, in the last decades, more asymptomatic children are diagnosed by serological screening programs. It is crucial to monitor the clinical trends of CD in order to improve awareness, for a proper approach and an early diagnosis.

## Data Availability

All data generated or analysed during this study are included in this published article.

## Abbreviations

CD: Celiac Disease
GFD: Gluten-Free-Diet
GIs: gastrointestinal symptoms
RAP: recurrent abdominal pain
EIs: extra-intestinal symptoms
FTT: failure to thrive
GER: gastroesophageal reflux
